# Elevated HbA1c is not associated with recurrent venous thromboembolism in the elderly, but with all-cause mortality– the SWEETCO 65+ study

**DOI:** 10.1038/s41598-020-59173-2

**Published:** 2020-02-12

**Authors:** Alexandra Mathis, Lukas Villiger, Martin F. Reiner, Michael Egloff, Hans Ruedi Schmid, Simona Stivala, Andreas Limacher, Marie Mean, Drahomir Aujesky, Nicolas Rodondi, Anna Angelillo-Scherrer, Marc Righini, Daniel Staub, Markus Aschwanden, Beat Frauchiger, Joseph Osterwalder, Nils Kucher, Christian M. Matter, Martin Banyai, Oliver Hugli, Juerg H. Beer

**Affiliations:** 1 DiaMon Institute, Baden-Dättwil, Switzerland; 2 Department of Internal Medicine, Cantonal Hospital of Baden, Baden, Switzerland; 3 Center for Molecular Cardiology, University of Zurich, Schlieren, Switzerland; 4 Central Laboratory, Cantonal Hospital of Baden, Baden, Switzerland; 5 CTU Bern, and Institute of Social and Preventive Medicine (ISPM), University of Bern, Bern, Switzerland; 6 Division of General Internal Medicine, Bern University Hospital, Inselspital, University of Bern, Bern, Switzerland; 7 Division of Internal Medicine, Lausanne University Hospital, Lausanne, Switzerland; 8 Institute of Primary Health Care (BIHAM), University of Bern, Bern, Switzerland; 9 Department of Hematology and Central Hematology Laboratory, Bern University Hospital, Inselspital, University of Bern, Bern, Switzerland; 10 Department of Bio Medical Research, University of Bern, Bern, Switzerland; 11 Division of Angiology and Hemostasis, Geneva University Hospital, Geneva, Switzerland; 12 Division of Angiology, Basel University Hospital, Basel, Switzerland; 13 Department of Internal Medicine, Cantonal Hospital of Frauenfeld, Frauenfeld, Switzerland; 14 Cantonal Hospital of St. Gallen, St. Gallen, Switzerland; 15 Division of Angiology, Zurich University Hospital, Zurich, Switzerland; 16 Department for Cardiology, University Heart Center, Zurich University Hospital, Zurich, Switzerland; 17 Division of Angiology, Cantonal Hospital of Lucerne, Lucerne, Switzerland; 18 Emergency Department, Lausanne University Hospital, Lausanne, Switzerland

**Keywords:** Endocrinology, Medical research

## Abstract

The association of glycated hemoglobin (HbA1c) with venous thromboembolism (VTE) and death in the elderly is unknown. In the SWEETCO 65+ study we analyzed prospectively a Swiss Cohort of Elderly Patients with Venous Thromboembolism (SWITCO 65+). 888 patients were enrolled for the SWEETCO 65+ analysis. HbA1c was determined at baseline and divided into three categories (HbA1c < 5.7%, normal range; 5.7–6.49%, pre-diabetic range; and >6.5%, diabetic range). Median follow-up was 2.5 years. The primary endpoint was recurrent VTE. Secondary endpoints included all-cause mortality and major bleeds. The total prevalence of diabetes was 22.1%. The risk of recurrent VTE was similar in patients with HbA1c with pre-diabetes (adjusted subhazard ratio (aSHR) 1.07 [0.70 to 1.63]) and diabetes (aSHR 0.73 [0.39 to 1.37]) as compared to those with a HbA1c in the normal range. However, a HbA1c ≥ 6.5% (median IQ range 7.0 [6.70;7.60]) was significantly associated with a higher risk of all-cause mortality (adjusted hazard ratio [aHR] 1.83 [1.21 to 2.75]). In summary we found no association between HbA1c and major bleeding. Elevated HbA1c levels are not associated with recurrent VTE but with increased all-cause mortality in an elderly population with acute VTE.

## Introduction

Venous thromboembolism (VTE) is a major cause of cardiovascular mortality^1^. Recurrence of VTE occurs in 29% of patients within 5 years after suspending anticoagulant treatment^2^, and life-long anticoagulation is recommended in unprovoked or recurrent VTE after taking the bleeding risk into consideration^3^. Anticoagulation is associated with serious side effects, particularly major bleeding, and increased health-care costs^4,5^. Therefore, efforts are required to identify and reduce risk factors for recurrent VTE in order to optimize treatment strategies for patients with VTE.

Patients with VTE are at increased risk of stroke and myocardial infarction^6–8^; hence, shared risk factors for arterial and venous thrombogenesis are currently discussed including diabetes mellitus (DM), hypertension, tobacco use, obesity, and dyslipidemia^9–11^. All provoke inflammation, hypercoagulability and endothelial injury, finally causing arterial and venous thromboembolism^12–28^. Experimental studies have shown higher platelet (re)activity^16,29–31^ and increased levels of clotting factors including fibrinogen, factor V, VII, VIII, X, XI, XII, as well as kallikrein and von Willebrand factor in patients with hyperglycemia^18,32–34^. This prothrombotic condition in combination with hypofibrinolysis^18,32–35^ observed in diabetic patients leads to a hemostatic imbalance, which might contribute to a higher risk for VTE and recurrent VTE. However, the association of DM and VTE in epidemiological studies is controversial. A meta-analysis showed an increased risk for VTE in patients with a history of DM^36^ whereas another meta-analysis found no association between DM and VTE^37,38^, concluding that the increased risk for VTE associated with DM mainly results from confounders rather than from intrinsic effects of DM on venous thrombotic risk^38^.

Studies which investigated the association between DM and recurrent VTE are scarce. One study found a significant association and another found a positive relationship between diagnosis of DM and recurrent VTE^39,40^. Importantly, DM was mostly defined by medical chart review, self-reported DM, elevated non-fasting or fasting glucose levels or seldom with an oral glucose tolerance test. In none of these studies glycated hemoglobin (HbA1c) was measured.

Glycated hemoglobin (HbA1c) reflects the average blood glucose levels over the last 2 to 3 months^41–43^. Large clinical trials indicate a reduction in microvascular and to a lesser extent in macrovascular complications if HbA1c values <7% in combination with treatment of other cardiovascular risk factors are achieved^44–46^. Therefore, DM as characterized by HbA1c levels might be a valid marker for predicting recurrent VTE risk.

In the current study, we investigated the association of HbA1c levels with recurrent VTE, all-cause mortality, and major bleedings in patients aged ≥65 years with acute VTE. We hypothesized that recurrent VTE and all-cause mortality would be increased in patients with HbA1c ≥ 6.5%.

## Methods

### Study population

The SWEETCO 65+ study was performed as part of the Swiss Cohort of Elderly Patients with Venous Thromboembolism (SWITCO 65+), a prospective multicenter cohort study to explore the associations of clinical and biological factors as well as processes of care with short- and long-term medical outcomes and quality of life in elderly patients with acute VTE^47^. The central ethics committee in Bern and the ethics committee of northeastern Switzerland approved the study. All research was performed in accordance with relevant guidelines. All study participants provided signed informed consent. From September 2009 to March 2012 1,863 in- and outpatients aged ≥65 years with objectively diagnosed, symptomatic acute VTE (deep vein thrombosis and/or pulmonary embolism) from all five Swiss university hospitals and four high-volume non-university teaching hospitals were screened.

DVT was defined by clinical assessment and by objective confirmation by duplex flow pattern, computed tomography or magnetic resonance imaging venography^47^. Symptomatic PE was defined as a positive spiral computed tomography or pulmonary angiography, a high-probability ventilation-perfusion scan, or proximal DVT in patients with acute chest pain, new or worsening dyspnea, hemoptysis or syncope^47^.

A total of 1863 patients were screened. After removal of 860 patients (398 patients refused consent; 462 patients had at least one of the following exclusion criteria: inability to provide informed consent, follow-up not possible, insufficient ability to speak German or French, thrombosis at different site than lower limb or catheter-related thrombosis), 1,003 patients were enrolled in the SWITCO 65+ study. An additional 115 patients were excluded from analysis (denying use of data [n = 8], early withdrawal [n = 4], no biosample taken [n = 89], missing HbA1c result [n = 14]), leaving a final study sample of 888 patients for the current analysis (Supplemental Fig. 1).

### Data collection

Trained study nurses prospectively collected baseline demographic information (age and gender), comorbidities (cancer, DM, BMI, chronic renal and liver disease, recent myocardial infarction and history of stroke and bleeding, recent immobilization), laboratory findings (hemoglobin, creatinine and platelets), VTE-related treatment before and after the index event (low-molecular-weight heparin, unfractionated heparin, fondaparinux, danaparoid) and concomitant antiplatelet therapy using standardized data collection forms for all enrolled patients. Known diabetes was defined by patient reports in addition to medical records.

### Determination of HbA1c levels

HbA1c was determined at baseline using the Tina-quant Hemoglobin A1c III test from Roche/Hitachi, a turbidimetric inhibition immunoassay (TINIA) for the *in vitro* determination of hemoglobin A1c in whole blood according to the instructions of the manufacturer. Results are expressed as % HbA1c according to the Diabetes Control and Complications Trial assay (DCCT) and to the National Glycohemoglobin Standardization Program (NGSP)^48^. Treating physicians were unaware of the HbA1c levels.

### HbA1c-categories

HbA1c was divided into three categories (HbA1c < 5.7%, normal range; HbA1c 5.7–6.49%, pre-diabetes range; and HbA1c > 6.5%, diabetes range) according to current guidelines for the definition of prediabetes by HbA1c of 5.7–6.49% and diabetes by HbA1c ≥ 6.5%^49–51^. Achieving HbA1c values <6.5% with pre-existing diabetes is possible, nevertheless, in the same patients increased HbA1c may have existed before resulting in an elevated cardiovascular risk. Otherwise HbA1c values ≥6.5% may reflect undiagnosed DM. Therefore, in a second analysis, we stratified patients into two subgroups with known diabetes and no history of diabetes. Patients were grouped into the same HbA1c-categories as described above. As an HbA1c level of <5.7% is unusual for diabetic patients and might indicate aggressively treated disease with frequent hypoglycemias, we took the HbA1c category pre-diabetes range as reference group for this second analysis of patients with known diabetes.

### Endpoints

We evaluated outcomes using patient or proxy interviews, interview of the patient’s primary care physician, and/or hospital chart review. A committee of three blinded clinical experts determined all outcomes and classifies the cause of all deaths as definitely due to PE, possibly due to PE, due to major bleeding, or due to another cause^52,53^. Final classifications were made on the basis of the full consensus of this committee.

Our primary endpoint was recurrent VTE. New fatal or new non-fatal PE, or new DVT (proximal and/or distal) were considered as recurrent VTE. The following criteria counted for the diagnosis of recurrent VTE during follow-up: Abnormal results on ultrasonography for DVT and for PE, new intraluminal defects on CT or angiography or ventilation-perfusion lung scan showing a high-probability pattern with new perfusion defects. A new proximal DVT in combination with new PE symptom(s) (syncope, chest pain, shortness of breath) was defined as recurrent PE^47^.

Our secondary endpoints included mortality and major bleeding. Major bleeding was defined as symptomatic bleeding in a critical organ (intracranial, intraspinal, intraocular, retroperitoneal, intraarticular, pericardial or intramuscular with compartment syndrome), bleeding with a reduction of haemoglobin ≥20 g/L, or bleeding leading to the transfusion ≥2 units of packed red blood cells^47^.

### Statistical analyses

We compared baseline characteristics across HbA1c groups using the chi-squared test and the non-parametric Wilcoxon rank-sum test or Kruskal-Wallis rank test as appropriate. We estimated and compared the cumulative incidence of VTE recurrence, death, and major bleeding by HbA1c group using the Kaplan-Meier method and the log-rank test.

Associations between HbA1c groups and the time to a first VTE recurrence and major bleeding were assessed by competing risk regression, accounting for non-PE-related and non-bleeding-related death, respectively, as a competing event, according to the method of Fine and Gray^54^. The method yields subhazard ratios (SHR) with corresponding 95%CIs and P values for the failure event of primary interest. For all-cause mortality, an ordinary Cox-regression with robust standard errors was calculated. Patients were censored when they were lost to follow-up (n = 3), withdrew their consent (n = 57), or completed the study without experiencing the event of interest or the competing event, if applicable.

Similar to our previous publication^55^ risk factors that had formerly been shown to be associated with VTE recurrence, all-cause mortality and bleeding were used to adjust the models. VTE recurrence was adjusted for prior VTE, provoked VTE, gender, age, active cancer, periods of anticoagulation as a time-varying covariate and BMI. Death was adjusted for age, gender, heart failure, immobilization, active cancer, chronic lung disease, low blood pressure, anemia, overt PE, history of major bleeding, high pulse, high creatinine, BMI and periods of anticoagulation as a time-varying covariate. Major bleeding was adjusted for age, active cancer, overt PE, anemia, history of major bleeding, high creatinine, antiplatelet therapy and periods of anticoagulation as a time-varying covariate.

Minimal adjustment was done in patients with a history of DM due to low event numbers. Similar to our previous study^55^ age, active cancer, and periods of anticoagulation as a time-varying covariate was used for adjust for recurrent VTE. Death was adjusted for active cancer, age, chronic lung disease, BMI, heart failure and periods of anticoagulation as a time-varying covariate. For major bleeding was adjusted for antiplatelet therapy, periods of anticoagulation as a time-varying covariate age and history of major bleeding.

Similar to our previous publication^55^ missing values in covariates used for adjustment were rare (<8%) and thus assumed to be normal or absent. All analyses were done using Stata 14 (Stata Corporation, College Station, Texas).

### Ethics approval and consent to participate

The central ethics committee in Bern and the ethics committee of northeastern Switzerland approved the study. All research was performed in accordance with relevant guidelines. All study participants provided signed informed consent; the full description of the study design has been registered on clinical trials.gov identifier NCT00973596.

### Consent for publication

The journal Primary and Hospital Care has approved the use of the previous published data and figures.

## Results

### Study population

The mean age of patients was 75 years and 46% were female. Patients’ baseline characteristics were comparable across HbA1c categories with the following exceptions (Table 1): BMI differed significantly between the HbA1c categories. Patients with a normal HbA1c had a significantly higher prevalence of provoked index VTE, major surgery and immobilization during the last 3 months compared to other categories. Patients with HbA1c in diabetes range had significantly higher prevalence of arterial hypertension, chronic renal disease and concomitant antiplatelet therapy and those with HbA1c in pre-diabetes range had less often a history of major bleeding and anemia compared with the other categories.

**Table 1:** Baseline characteristics by HbA1c categories.

	All	Category normal range HbA1c <5.7%	Category pre-diabetes range HbA1c 5.7 to 6.49%	Category diabetes range HbA1c ≥6.5%	p-value
	n (%) or median (IQ-range)	n (%) or median (IQ-range)	n (%) or median (IQ-range)	n (%) or median (IQ-range)	
Total N	N = 888	N = 452	N = 300	N = 136	
Age	75.00 (69.00; 81.00)	74.00 (69.00; 80.75)	76.00 (70.00; 82.00)	75.00 (69.00; 81.00)	0.079
Female gender	408 (46%)	214 (47%)	137 (46%)	57 (42%)	0.533
BMI	26.60 (24.10; 29.80)	26.00 (23.65; 29.10)	27.00 (24.20; 30.10)	28.10 (24.80; 31.10)	<0.001
Overt PE	619 (70%)	309 (68%)	210 (70%)	100 (74%)	0.512
Prior VTE	254 (29%)	126 (28%)	86 (29%)	42 (31%)	0.793
Provoked index VTE	258 (29%)	159 (35%)	65 (22%)	34 (25%)	<0.001
Major surgery during the last 3 months	131 (15%)	94 (21%)	25 (8%)	12 (9%)	<0.001
Current oestrogen therapy during the last 3 months	27 (3%)	15 (3%)	7 (2%)	5 (4%)	0.661
Immobilization during the last 3 months	191 (22%)	116 (26%)	50 (17%)	25 (18%)	0.008
Active cancer	160 (18%)	90 (20%)	47 (16%)	23 (17%)	0.312
Known diabetes (as reported)	139 (16%)	26 (6%)	34 (11%)	79 (58%)	<0.001
History of major bleeding	89 (10%)	60 (13%)	16 (5%)	13 (10%)	0.002
Arterial hypertension	571 (64%)	270 (60%)	196 (65%)	105 (77%)	0.001
Chronic or acute heart failure	105 (12%)	47 (10%)	34 (11%)	24 (18%)	0.068
Cerebrovascular disease (stroke, TIA)	84 (9%)	40 (9%)	30 (10%)	14 (10%)	0.815
Chronic lung disease	124 (14%)	56 (12%)	41 (14%)	27 (20%)	0.087
Chronic renal disease	163 (18%)	73 (16%)	53 (18%)	37 (27%)	0.013
Anemia	346 (39%)	196 (43%)	94 (31%)	56 (41%)	0.002
Platelet count <150 G/l	131 (15%)	71 (16%)	42 (14%)	18 (13%)	0.625
Creatinine >107 umol/l	208 (23%)	86 (19%)	74 (25%)	48 (35%)	0.001
Heart rate > = 110 beats/min	85 (10%)	38 (8%)	33 (11%)	14 (10%)	0.487
Systolic blood pressure <100 mmHg	32 (4%)	13 (3%)	16 (5%)	3 (2%)	0.132
Polypharmacy	452 (51%)	225 (50%)	135 (45%)	92 (68%)	<0.001
Anticoagulation prior to index VTE	44 (5%)	24 (5%)	12 (4%)	8 (6%)	0.622
Type of initial parenteral anticoagulation					0.388
Low-molecular-weight heparin	435 (49%)	232 (51%)	140 (47%)	63 (46%)	
Unfractionated heparin	273 (31%)	142 (31%)	93 (31%)	38 (28%)	
Fondaparinux	149 (17%)	63 (14%)	55 (18%)	31 (23%)	
Danaparoid	1 (0%)	1 (0%)	0 (0%)	0 (0%)	
None	30 (3%)	14 (3%)	12 (4%)	4 (3%)	
Initial Vitamin K antagonist therapy	773 (87%)	390 (86%)	261 (87%)	122 (90%)	0.581
Concomitant antiplatelet therapy	282 (32%)	131 (29%)	90 (30%)	61 (45%)	0.002
HbA1c [%]	5.70 (5.40; 6.10)	5.40 (5.20; 5.60)	6.00 (5.90; 6.20)	7.00 (6.70; 7.60)	

Values were missing in body mass index (1%), heart rate (2%), systolic blood pressure (2%), anaemia (6%), platelet count (6%) and creatinine (8%). Provoked venous thromboembolism was defined as presence of at least one of the following factors: major surgery, oestrogen therapy or immobilization during the last 3 months. Active cancer was defined as cancer that required therapie (surgery, chemotherapy, and/or radiotherapy) during the last 3 months. Major bleeding was defined as a fatal bleeding, a symptomatic bleeding in a critical organ (intracranial, intraspinal, intraocular, retroperitoneal, intraarticular, pericardial, or intramuscular with compartment syndrom), a bleeding with a reduction of hemoglobin ≥20 g/l, or a bleeding leading to the transfusion ≥2 units of packed red blood cells. Polypharmacy was defined as prescription of more than four different drugs. IQ = interquartile, PE = pulmonary embolism, TIA = transient ischemic attack, VTE = venous thromboembolism.

### Prevalence of diabetes and prediabetes

A history of diabetes was identified in 15.6% (n = 139) of the study population. After HbA1c determination, the prevalence of diabetes increased to 22.1% (n = 196). Only 6.1% (n = 54) of the study population had an HbA1c > 7.3% and HbA1c > 8% was seen in 2.9% only (n = 26). The prevalence of prediabetes in patients with no history of diabetes was high, namely 36.0% (n = 266) and 30% in the whole study population.

### Recurrent VTE

**Figure 1:**
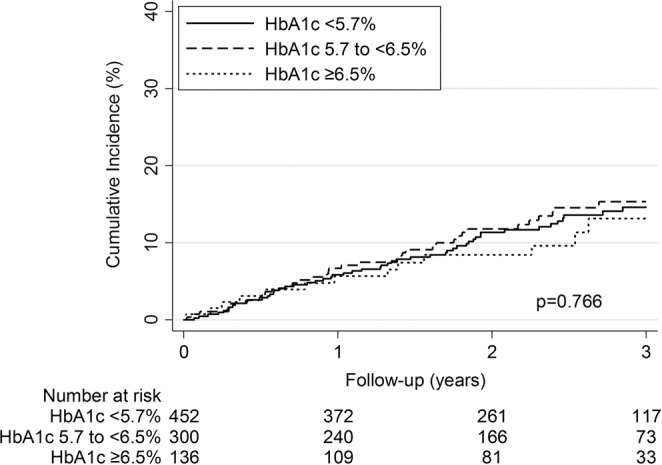
Kaplan-Meier estimates of cumulative VTE recurrence by HbA1c categories. The cumulative incidence of recurrent VTE did not differ between groups (log-rank p = 0.766).

Patients were followed for up to four years (median 2.5 years). After 3 years, the cumulative incidence of the recurrent VTE did not differ between groups (Fig. 1). During the whole follow-up, the risk for recurrent VTE in patients with HbA1c in pre-diabetes range (adjusted subhazard ratio [aSHR] 1.07 [0.70–1.63], p = 0.756) and diabetes range (aSHR 0.73 [0.39–1.37], p = 0.328) was comparable to patients with normal range HbA1c (Table 2). Likewise, in a subgroup analysis of patients with and without history of diabetes, the risk for recurrent VTE did not differ between HbA1c-categories (Table 2).

**Table 2:** Association of HbA1c categories with VTE recurrence.

	HbA1c group	HbA1c	n/N	Crude SHR (95%-CI)	p-value	Adjusted SHR (95%-CI)	p-value
All patients	normal range	<5.7%	56/452	1 (reference)		1 (reference)	
	pre-diabetes range	5.7 to <6.5%	40/300	1.11 (0.74 to 1.66)	0.617	1.07 (0.70 to 1.63)	0.756
	diabetes range	≥6.5%	13/136	0.75 (0.41 to 1.38)	0.353	0.73 (0.39 to 1.37)	0.328
Patients with history of diabetes	normal range	<5.7%	3/26	0.76 (0.19 to 2.98)	0.696	0.81 (0.22 to 3.00)	0.754
	pre-diabetes range	5.7 to <6.5%	6/34	1 (reference)		1 (reference)	
	diabetes range	≥6.5%	8/79	0.62 (0.22 to 1.75)	0.365	0.68 (0.24 to 1.92)	0.468
Patients without history of diabetes	normal range	<5.7%	53/426	1 (reference)		1 (reference)	
	pre-diabetes range	5.7 to <6.5%	34/266	1.08 (0.70 to 1.66)	0.731	1.03 (0.66 to 1.62)	0.884
	diabetes range	≥6.5%	5/57	0.67 (0.27 to 1.68)	0.393	0.70 (0.27 to 1.79)	0.454

VTE recurrence was adjusted for age, gender, active cancer, provoked VTE, prior VTE, body mass index and periods of anticoagulation as a time-varying covariate. CI = confidence interval, n = number of events, N = number of patients, SHR = sub-hazard ratio, VTE = venous thromboembolism.

### All-cause mortality

**Figure 2:**
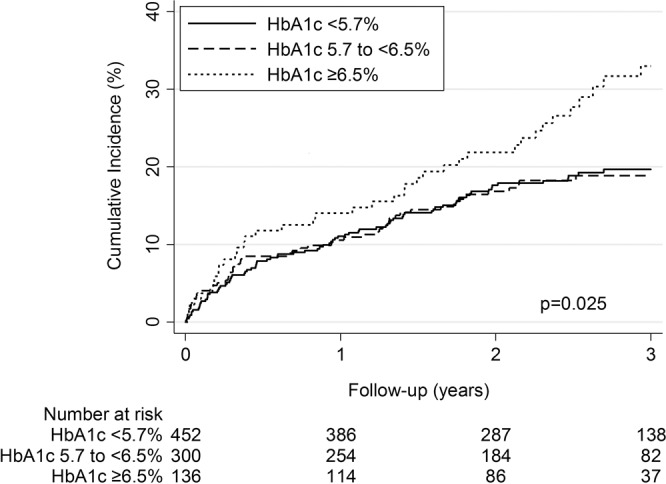
Kaplan-Meier estimates of cumulative mortality by HbA1c categories. The cumulative mortality was higher in patients with HbA1c ≥ 6.5% than in patients with lower HbA1c levels (log-rank p = 0.025).

**Figure 3:**
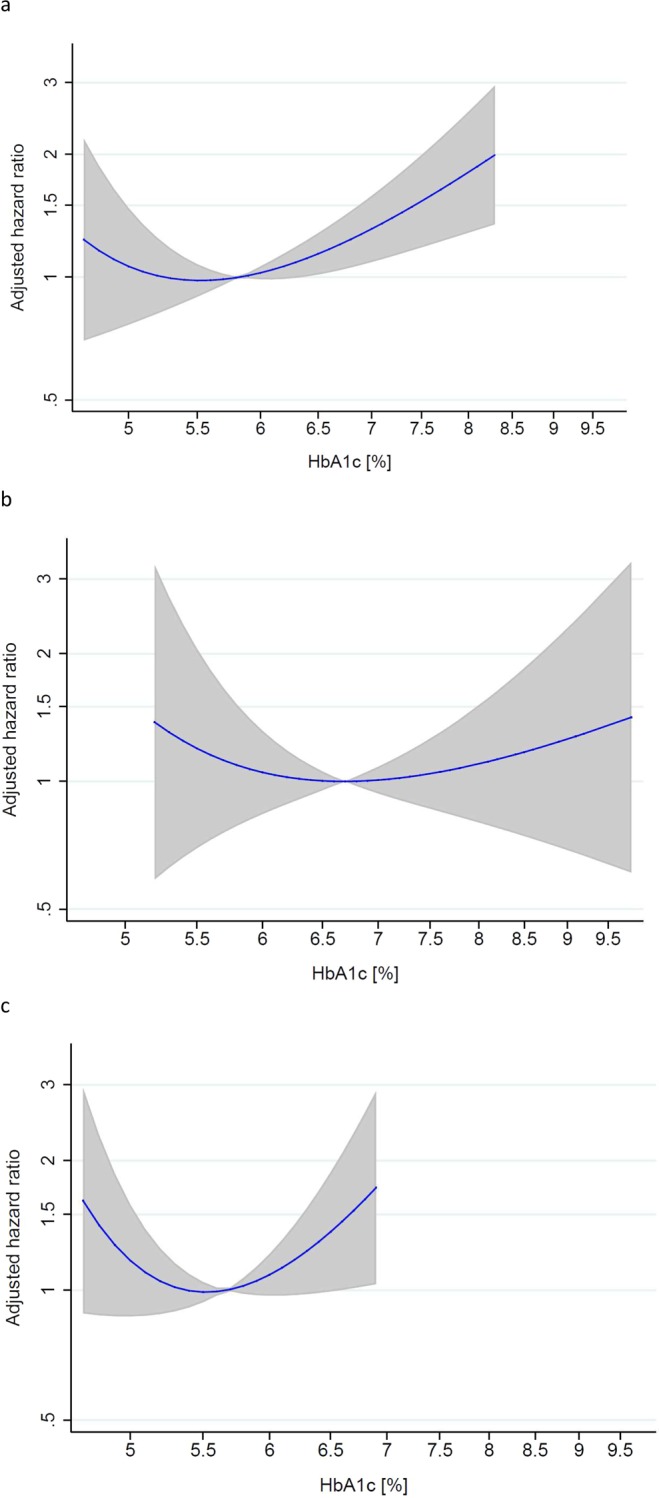
Relative hazards for mortality according to HbA1c values. Adjusted hazard ratios with 95% confidence intervals from a fractional polynomial Cox-proportional hazards model with robust standard errors. HbA1c values were used log-transformed and centered at the mean. Both axes are shown on a natural-log scale. (**a**) Relative hazards for mortality according to HbA1c values in all patients (N = 888). The relationship between continuous HbA1c and mortality is U-shaped (p = 0.002). The plot was truncated at the 2.5th and 97.5th percentile of HbA1c (4.7% and 8.3%, respectively). (**b**) Relative hazards for mortality according to HbA1c values in subgroup of patients with history of diabetes (N = 139). The relationship between continuous HbA1c and mortality is slightly U-shaped (p = 0.63) in patients with history of diabetes. The plot was truncated at the 5th and 95th percentile of HbA1c (5.2% and 9.8%, respectively). (**c**) Relative hazards for mortality according to HbA1c values in subgroup of patients without history of diabetes (N = 749). The relationship between continuous HbA1c and mortality is U-shaped (p = 0.064). The plot was truncated at the 2.5th and 97.5th percentile of HbA1c (4.7% and 6.9%, respectively).

Patients with HbA1c in diabetes range had a significantly higher mortality risk compared to patients with HbA1c in normal range (aHR 1.83 [1.21–2.75], p = 0.004), whereas we did not observe a higher risk for mortality in patients with HbA1c in pre-diabetes range (aHR 1.17 [0.82–1.69], p = 0.388) (Fig. 2 and Table 3). In a secondary analysis, we studied a possible non-linear relationship between continuous HbA1c values and mortality in a fractional polynomial Cox-proportional hazards model. This model showed a U-shaped relationship between HbA1c and relative hazards of mortality (p = 0.002, Fig. 3a). This result was confirmed when assuming a quadratic relationship by using a linear and quadratic term for HbA1c in a Cox-proportional hazards model (p = 0.02).

**Table 3:** Association of HbA1c categories with mortality.

	HbA1c group	HbA1c	n/N	Crude HR (95%-CI)	p-value	Adjusted HR (95%-CI)	p-value
All patients	normal range	<5.7%	90/452	1 (reference)		1 (reference)	
	pre-diabetes range	5.7 to <6.5%	55/300	0.94 (0.67 to 1.32)	0.741	1.17 (0.82 to 1.69)	0.388
	diabetes range	≥6.5%	41/136	1.57 (1.09 to 2.25)	0.015	1.83 (1.21 to 2.75)	0.004
Patients with history of diabetes	normal range	<5.7%	8/26	2.24 (0.74 to 6.82)	0.154	1.44 (0.45 to 4.60)	0.537
	pre-diabetes range	5.7 to <6.5%	6/34	1 (reference)		1 (reference)	
	diabetes range	≥6.5%	25/79	2.04 (0.81 to 5.14)	0.130	1.39 (0.53 to 3.65)	0.498
Patients without history of diabetes	normal range	<5.7%	82/426	1 (reference)		1 (reference)	
	pre-diabetes range	5.7 to <6.5%	49/266	1.00 (0.70 to 1.43)	0.991	1.20 (0.81 to 1.76)	0.366
	diabetes range	≥6.5%	16/57	1.51 (0.88 to 2.58)	0.135	2.12 (1.16 to 3.90)	0.015

Mortality was adjusted for age, gender, active cancer, immobilization, heart failure, chronic lung disease, overt pulmonary embolism, history of major bleeding, high pulse, low blood pressure, anemia, high creatinine, body mass index and periods of anticoagulation as a time-varying covariate. HR = hazard ratio, n = number of events, N = number of patients.

A total of 186 deaths were observed: 61 deaths (32.8%) were attributable to cancer, 7 (3.8%) PE related, 28 [15.1%] possibly PE related, 16 (8.6%) to sepsis, 14 (7.5%) to infection, 12 (6.5%) to bleeding, 11 (5.9%) to left ventricular failure, 6 (3.2%) to pulmonary causes other than PE, 3 (1.6%) to acute coronary syndrome, 2 (1.1%) to stroke, 3 (1.6%) to suicide, 4 (2.2%) to others, and 19 (10.2%) to unknown causes (Supplemental Table 1).

In the subgroup analysis of patients without a history of diabetes, we observed similar results. HbA1c in diabetes range was significantly associated with a higher risk of mortality (aHR 2.12 [1.16–3.90] p = 0.015). The risk of mortality was similar between patients with pre-diabetes and patients with normal HbA1c levels (aHR 1.20 [0.81–1.76] p = 0.366) (Table 3). Causes of death are shown in Supplemental Table 1.

In the subgroup analysis of patients with a history of diabetes, the risk of all-cause mortality was higher in patients with HbA1c in normal range (aHR 1.44 [0.45–4.60] p = 0.537) and HbA1c in diabetes range (aHR 1.39 [0.53–3.65], p = 0.498), as compared to individuals with HbA1c in pre-diabetes range. Interestingly, all deaths in patients with known diabetes and HbA1c levels <5.7% occurred within the first year of follow up. Causes of death are shown in Supplemental Table 1.

In a secondary analysis, we determined relative hazards for mortality according to HbA1c values. The fractional polynomial Cox-proportional hazards model showed a U-shaped relationship between continuous HbA1c and mortality (Fig. 3a–c).

### Major bleeding

The risk of major bleeding was not greater in patients with HbA1c in pre-diabetes range (aSHR 0.82 [0.54–1.23] p = 0.328) and diabetes range (aSHR 0.67 [0.39–1.18] p = 0.168) compared to patients with HbA1c in normal range. Similar results were obtained in the subgroup analysis (data not shown).

## Discussion

This prospective multicenter cohort study investigated the association of HbA1c with recurrent VTE, all-cause mortality, and major bleeding in elderly patients with acute VTE. The prevalence of diabetes and prediabetes according to HbA1c levels was very high, amounting to 22.1% and 30.0%, respectively. During the follow-up, 109 patients (12%) experienced a recurrent VTE and 186 patients died (21%). We found no association between HbA1c levels and recurrent VTE and patients with HbA1c in pre-diabetes or diabetes range despite a higher baseline unprovoked VTE proportion (prediabetes range +13%, diabetes range +10%). However, our results showed that an HbA1c ≥ 6.5% was significantly associated with a higher mortality risk in both patients with and without a history of diabetes confirming results of previous studies^56,57^. Most interestingly, we found a flat U-shaped relationship between continuous HbA1c values and mortality, as had been observed earlier, however in a much younger population^58,59^. Lastly, we found that HbA1c levels were not associated with major bleeding.

Our results regarding VTE recurrences are in contrast with recent studies, which found a positive relationship between diabetes and the incidence of recurrent VTE^39,40^. These studies may have possible bias by defining DM according to medical chart review and not by measuring HbA1c. One important reason for the lack of association between HbA1c and recurrent VTE in our study is the fact that the diabetic patients were well controlled; only 6% (n = 54) of participants had an HbA1c > 7.3% and only 3% (n = 26) had an HbA1c > 8%. We cannot exclude an association of poorly controlled diabetes exhibiting very high HbA1c levels with recurrent VTE, which should be motivation enough to achieve better diabetes control.

We found a U-shaped relationship between continuous HbA1c values and all-cause mortality, demonstrating that patients with elevated HbA1c as well as patients with low HbA1c values are at higher mortality risk. Due to the very low proportion of high HbA1c, i.e. >8% (n = 26) in patients with history of diabetes, our HbA1c nadir with the lowest mortality rate (6.7%) was lower than in another study (HbA1c nadir 7.5–7.9%)^58^, again suggesting excellent diabetes control. The HbA1c nadir was 5.5% in our patients without history of diabetes and therefore comparable to other articles (HbA1c nadir 5.3%)^59^.

In the subgroup of patients with a history of diabetes, HbA1c in diabetes range and HbA1c in normal range were positively associated with higher mortality rates taking the HbA1c category pre-diabetes range as the reference. The higher mortality in the HbA1c < 5.7% group corresponds to the observation of others^60^. A possible explanation is an association of lower HbA1c values with severe illness or (overly) tight glycemic control with recurrent hypoglycemias. Hypoglycemia decreases myocardial perfusion^61^ and leads to prolonged QT in patients with diabetes type 1^62^. Such findings may explain the association (and pathophysiology) of hypoglycemia and increased cardiovascular mortality including sudden deaths in susceptible individuals.

The prevalence of diabetes (22.1%) and prediabetes (30.0%) was remarkably high in this elderly population with acute VTE and slightly higher compared to the percentages found in the CoLaus study, a population based study from Lausanne, Switzerland^63^. In the CoLaus study the prevalence of type 2 DM (defined as fasting plasma glucose >7 mmol/l and/or antidiabetic treatment) in the age group 65–75 years was 18.9%. In the KORA Augsburg study, a population based study in the southern part of Germany^64^, prevalence of DM was 22.4% in the age group 65–74 years and therefore comparable to our observation. Even higher diabetes prevalence was found in the US with 26% in the general population aged 65 years and older^65^.

The CoLaus study found that roughly one third were newly diagnosed diabetics after determination of increased plasma glucose levels. Likewise, we found that 29.1% of diabetic patients were newly diagnosed after HbA1c determination in our study population.

Our study has several limitations. The number of primary endpoint events in the subgroup of patients with history of diabetes was relatively low. HbA1c values have been measured at baseline and may have changed over the years of follow-up. Information on additional biological factors which may have influenced HbA1c levels^66^, such as conditions with rapid red cell turnover (hemoglobinopathies or transfusion) are lacking, albeit in most instances unlikely to be present. Likewise, we had no information on therapy and duration of DM. Known diabetes was defined by patient reports in addition to medical records; therefore, we cannot exclude that some patients have falsely been allocated to the group of “patients without a history of diabetes”. Furthermore, HbA1c level is age dependent. Despite similar blood glucose levels, HbA1c levels in participants aged >70 years were approximately 0.4 percentage point higher than in those <40 years of age in the Framingham study^67^. Nevertheless, to date, no age-specific diagnostic criteria or adaptations of any normal HbA1c values exist. The current study focuses exclusively on high risk patients above 65 years of age with acute VTE and therefore the data cannot be extrapolated to the general population. Despite extensive adjustments, we cannot exclude residual confounding. And finally, due to the relatively low number of elevated HbA1c values above 8%, our study does not exclude a potential association of poorly controlled diabetes with recurrent VTE.

In summary, we found that diabetes does not appear be an independent predictor for recurrent VTE in elderly patients with VTE. Likewise, HbA1c levels were not associated with major bleedings. However, we found a U-shaped relationship of HbA1c levels with all-cause mortality in elderly patients with acute VTE with the lowest mortality rate at an HbA1c level of 6.7% for patients with history of diabetes and 5.5% for patients without history of diabetes.

## Supplementary information

Dataset 1.

## Data Availability

The datasets used and/or analyzed during the current study are available from the corresponding author upon reasonable request.
