# Supplementary material for: Elevated HbA1c is not associated with recurrent venous thromboembolism in the elderly, but with all-cause mortality– the SWEETCO 65+ study
Source: Sci Rep. 2020 Feb 12;10:2495. doi: 10.1038/s41598-020-59173-2 (PMC7016100; doi:10.1038/s41598-020-59173-2)
Supplement: Supplementary file 1 — Dataset 1. [file 41598_2020_59173_MOESM1_ESM.docx]

**Elevated HbA1c is not associated with recurrent venous thromboembolism in the elderly, but with all-cause mortality– the SWEETCO 65+ study**

Alexandra Mathis*, Lukas Villiger, MD^1^, Martin F. Reiner, MD, PhD^2,3^, MD^1^, Michael Egloff, MD^2^, Hans Ruedi Schmid^4^, Simona Stivala, MD^3^, Andreas Limacher, PhD^5^, Marie Mean, MD^6,7^, Drahomir Aujesky MD, MSc^6^, Nicolas Rodondi, MD, MAS^6,8^, Anna Angelillo-Scherrer, MD^9,10^, Marc Righini, MD^11^, Daniel Staub, MD^12^, Markus Aschwanden, MD^12^, Beat Frauchiger, MD^13^, Joseph Osterwalder, MD^14^, Nils Kucher, MD^15^, Christian M. Matter, MD^3,16^, Martin Banyai, MD^17^, Oliver Hugli, MD, MPH^18^, Juerg H. Beer, MD^2,3^

^1^DiaMon Institute, Baden-Dättwil, Switzerland ^2^Department of Internal Medicine, Cantonal Hospital of Baden, Baden, Switzerland ^3^Center for Molecular Cardiology, University of Zurich, Schlieren, Switzerland ^4^Central Laboratory, Cantonal Hospital of Baden, Baden, Switzerland ^5^CTU Bern, and Institute of Social and Preventive Medicine (ISPM), University of Bern, Bern, Switzerland ^6^Division of General Internal Medicine, Bern University Hospital, Inselspital, University of Bern, Bern, Switzerland ^7^Division of Internal Medicine, Lausanne University Hospital, Lausanne, Switzerland ^8^Institute of Primary Health Care (BIHAM), University of Bern, Bern, Switzerland ^9^Department of Hematology and Central Hematology Laboratory, Bern University Hospital, Inselspital, University of Bern, Bern, Switzerland ^10^Department of Bio Medical Research, University of Bern, Bern, Switzerland ^11^Division of Angiology and Hemostasis, Geneva University Hospital, Geneva, Switzerland ^12^Division of Angiology, Basel University Hospital, Basel, Switzerland ^13^Department of Internal Medicine, Cantonal Hospital of Frauenfeld, Frauenfeld, Switzerland ^14^Cantonal Hospital of St. Gallen, St. Gallen, Switzerland ^15^Division of Angiology, Zurich University Hospital, Zurich, Switzerland ^16^Department for Cardiology, University Heart Center, Zurich University Hospital, Zurich, Switzerland ^17^Division of Angiology, Cantonal Hospital of Lucerne, Lucerne, Switzerland ^18^Emergency Department, Lausanne University Hospital, Lausanne, Switzerland

**Supplementary Figures**

**Suppl. Figure 1.



 Flow chart summarizing the enrollment of subjects for the Sweetco 65+ study**

**Suppl. Figure 2. Kaplan-Meier estimates of cumulative mortality by HbA1c-categories in patients with history of diabetes**

The cumulative mortality was comparable in all three patient groups (log-rank p=0.389). Notably, in patients with HbA1c<5.7%, all deaths occurred during the first year.

**Suppl. Figure 3. Kaplan-Meier estimates of cumulative mortality by HbA1c-categories in patients without history of diabetes**

The cumulative mortality was higher in patients with HbA1c ≥6.5% than in patients with lower HbA1c levels (log-rank p=0.177).

**Suppl. Figure 4. Kaplan-Meier estimates of cumulative major bleeding by HbA1c-categories**

The cumulative incidence of major bleeding did not differ between groups (log-rank p=0.527).

**Supplemental Figure 1. Enrollment of subjects for the Sweetco 65+ study**

Patients not eligible (n=462)

Thrombosis at a different site than lower limb (n=21)

Catheter-related thrombosis (n=7)

Insufficient spoken ability in German or French (n=51)

Inability to provide informed consent (n=285)

Follow-up not possible (n=192)

Screened (n=1863)

Patients who did not consent (n=398)

Enrolled (n=1003)

Patients excluded from analysis (n=115)

Denying use of data (n=8)

Early withdrawal (n=4)

No biosample taken (n=89)

No HbA1c result (n=14)

Evaluation of baseline HbA1c in 888 patients

Association with recurrent venous thromboembolism, mortality and major bleeding

History of diabetes

(n= 139)

no History of diabetes (n=749)

3 HbA1c-groups

<5.7%

5.7 to <6.5%

≥6.5%

3 HbA1c-groups

<5.7%

5.7 to <6.5%

≥6.5%

3 HbA1c-groups

<5.7%

5.7 to <6.5%

≥6.5%

**Supplemental Figure 2. Mortality by HbA1c-categories in patients with history of diabetes**

**
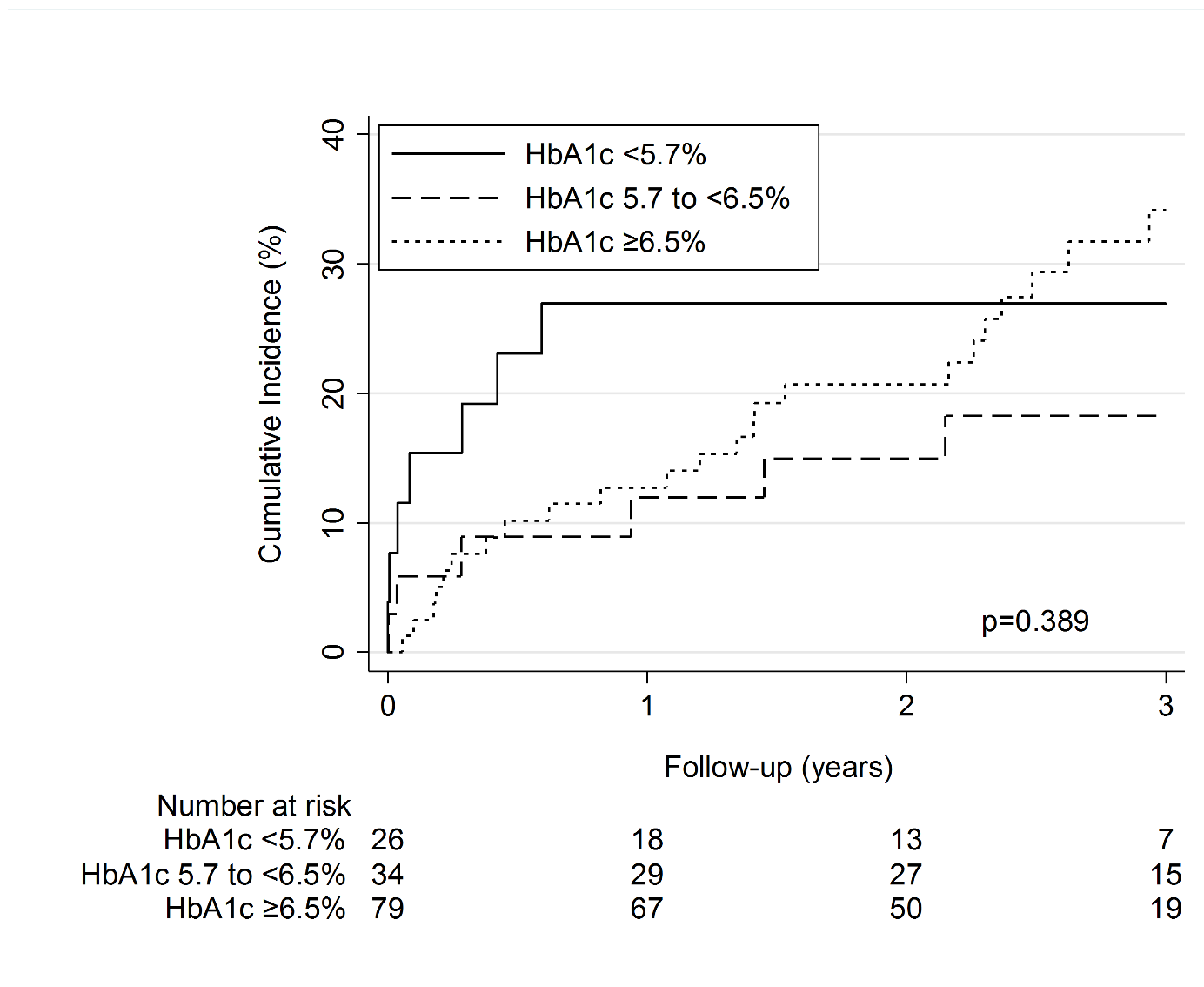
**

**Supplemental Figure 3. Mortality by HbA1c-categories in patients without history of diabetes**


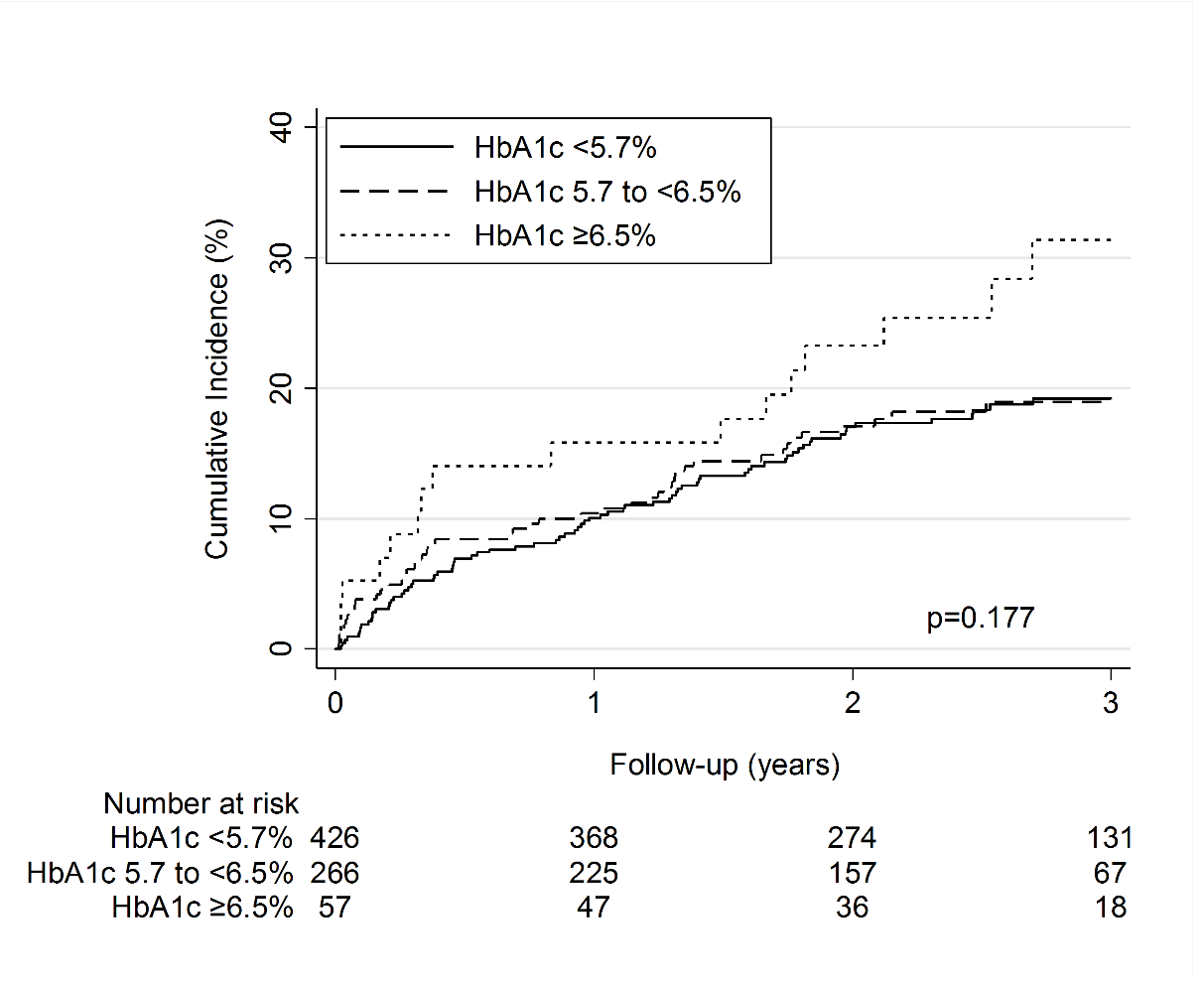


**Supplemental Figure 4.** **Major bleeding by HbA1c-categories**

**Supplemental Table 4. Causes of death by diabetes status known at baseline**

|  | All deaths | History of Diabetes | No History of Diabetes |
| --- | --- | --- | --- |
|  | N = 186 | N = 39 | N = 147 |
| Death cause |  |  |  |
| PE-related | 7 (3.8%) | 3 (7.7%) | 4 (2.7%) |
| Possibly PE-related | 28 (15.1%) | 4 (10.3%) | 24 (16.3%) |
| Bleeding | 12 (6.5%) | 2 (5.1%) | 10 (6.8%) |
| Acute coronary syndrom | 3 (1.6%) | 1 (2.6%) | 2 (1.4%) |
| Stroke | 2 (1.1%) | 0 (0.0%) | 2 (1.4%) |
| Left ventricular failure | 11 (5.9%) | 3 (7.7%) | 8 (5.4%) |
| Cancer | 61 (32.8%) | 7 (17.9%) | 54 (36.7%) |
| Infection | 14 (7.5%) | 3 (7.7%) | 11 (7.5%) |
| Sepsis | 16 (8.6%) | 5 (12.8%) | 11 (7.5%) |
| Pulmonary causes other than PE | 6 (3.2%) | 2 (5.1%) | 4 (2.7%) |
| Suicide | 3 (1.6%) | 1 (2.6%) | 2 (1.4%) |
| Other | 4 (2.2%) | 2 (5.1%) | 2 (1.4%) |
| Unknown | 19 (10.2%) | 6 (15.4%) | 13 (8.8%) |

PE = pulmonary embolism.
